# Factors correlated with poor glycemic control in patients with type 1 diabetes: result of a nationwide survey in Brazil

**DOI:** 10.1186/1758-5996-7-S1-A54

**Published:** 2015-11-11

**Authors:** Carine de Sousa Andrade Ribeiro, Carlos Antonio de Souza Teles Santos, Sandra S Moreira, Raimundo Celestino S Neves, Guilherme de Sousa Ribeiro, Edson Duarte Moreira

**Affiliations:** 1 Escola de Nutrição/Universidade Federal da Bahia, Salvador, Brazil

## Background

Diabetes increases the risk of cardiovascular and microvascular disease, particularly when not properly treated. Despite advances in diabetes management, inadequate glycemic control has been observed in 60% to 80% of patients. The objective of this study was to identify characteristics correlated with poor glycemic control in a large multicenter survey of Brazilian patients with type 1 diabetes.

## Materials and methods

We conducted a cross-sectional study in a consecutive sample of patients aged 18 yrs. or older with type 1 diabetes, attending health centers located in ten large cities in Brazil. Data on socio-demographics, treatment, and adherence to treatment were obtained by trained interviewers, using a standardized questionnaire. A peripheral blood sample was collected and HbA1c levels were measured by high-performance liquid chromatography in a central laboratory. Patients with HbA1c >7% were considered to have inadequate glycemic control. HbA1c was described by mean and standard deviation (SD). Bivariate linear regression analysis was performed to identify patients characteristics correlated with serum levels of HbA1c. Statistical significance was set at p<0.05.

## Results

Overall, 979 patients with type 1 diabetes were surveyed (mean age, 40 yrs.; female 63.8%). Mean level of HbA1c was 9.4% (SD: 2.22) and the prevalence of inadequate glycemic control was 89.6%. Higher HbA1c levels were correlated with socio-demographics (black race and low education), and treatment-related characteristics: last measurement of HbA1c >12 months ago, irregular finger glucose monitoring, irregular medical visits in the last 12 months, medical treatment performed by a non-specialist, non-participation in diabetes education programs, self-perception of inadequate insulin use, and self-perception of poor diet adherence.

## Conclusions

About 90% of the patients with type 1 diabetes in Brazil have inadequate glycemic control and are at increased risk for disease complications. Our findings may help guiding public health programs to improve glycemic control in this population.

